# Methodology to Create Auxin-Inducible Degron Tagging System to Control Expression of a Target Protein in Mammalian Cell Lines

**DOI:** 10.21769/BioProtoc.4923

**Published:** 2024-01-20

**Authors:** Amit Rahi, Deepika K. Sodhi, Christine B. Magdongon, Rajina Shakya, Dileep Varma

**Affiliations:** Department of Cell and Developmental Biology, Feinberg School of Medicine, Northwestern University, Chicago, IL 60611, USA

**Keywords:** Auxin-inducible degron (AID), Protein regulation, Auxin-induced degradation, CRISPR/Cas9, Protein tagging, Protein depletion, TIR1 receptor, Proteasomal degradation, Mitotic functions, CDT1

## Abstract

The auxin-inducible degron (AID) system is a versatile tool in cell biology and genetics, enabling conditional protein regulation through auxin-induced degradation. Integrating CRISPR/Cas9 with AID expedites tagging and depletion of a required protein in human and mouse cells. The mechanism of AID involves interactions between receptors like TIR1 and the AID tag fused to the target protein. The presence of auxin triggers protein ubiquitination, leading to proteasome-mediated degradation. We have used AID to explore the mitotic functions of the replication licensing protein CDT1. Swift CDT1 degradation via AID upon auxin addition achieves precise mitotic inhibition, revealing defects in mitotic spindle structure and chromosome misalignment. Using live imaging, we found that mitosis-specific degradation of CDT1 delayed progression and chromosome mis-segregation. AID-mediated CDT1 inhibition surpasses siRNA-based methods, offering a robust approach to probe CDT1’s mitotic roles. The advantages of AID include targeted degradation and temporal control, facilitating rapid induction and reversal of degradation—contrasting siRNA’s delayed RNA degradation and protein turnover. In summary, the AID technique enhances precision, control, and efficiency in studying protein function and regulation across diverse cellular contexts. In this article, we provide a step-by-step methodology for generating an efficient AID-tagging system, keeping in mind the important considerations that need to be adopted to use it for investigating or characterizing protein function in a temporally controlled manner.

Key features

• The auxin-inducible degron (AID) system serves as a versatile tool, enabling conditional protein regulation through auxin-induced degradation in cell biology and genetics.

• Integration of CRISPR/Cas9 knock-in technology with AID expedites the tagging and depletion of essential proteins in mammalian cells.

• AID’s application extends to exploring the mitotic functions of the replication licensing protein CDT1, achieving precise mitotic inhibition and revealing spindle defects and chromosome misalignment.

• The AID system and its diverse applications advance the understanding of protein function and cellular processes, contributing to the study of protein regulation and function.

**Graphical overview**


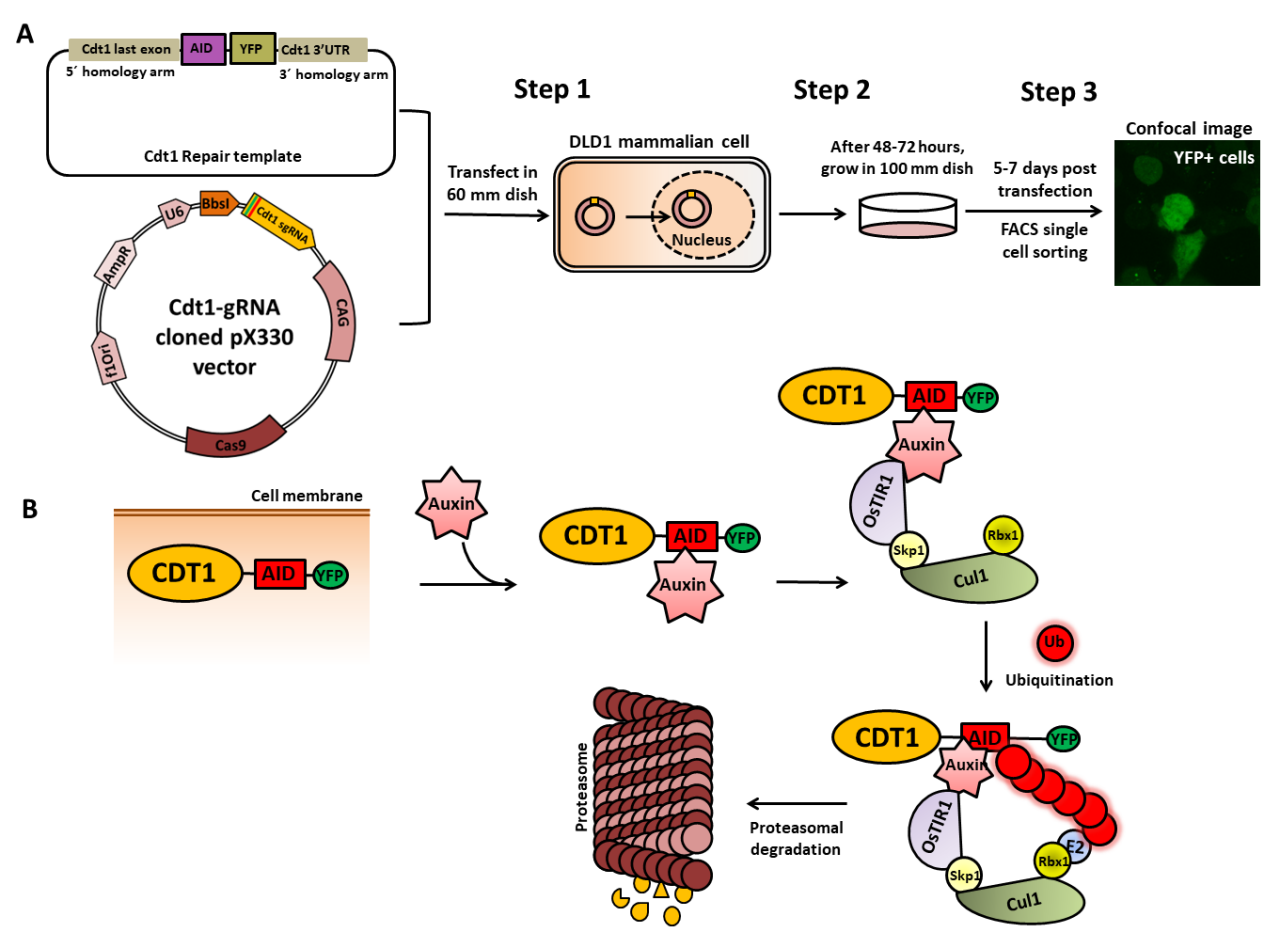


**Cdt1–auxin-inducible degron (AID) tagging workflow.** (A) Schematic of the cloned Cdt1 gRNA vector and the repair template generated to endogenously tag the Cdt1 genomic locus with YFP and AID at the C-terminal using CRISPR/CAS9-based genome editing. The two plasmids are transfected into DLD1-TIR1 stable cells, followed by sorting and scaling up of YFP-positive single cells. (B) The molecular mechanism of auxin-induced proteasome-mediated degradation of the target protein (CDT1) shown at the bottom of the figure is well worked out.

## Background

Conditional protein degradation is an invaluable approach to understand cellular function. Among the various techniques available, the auxin-inducible degron (AID) system stands out. AID is a versatile molecular tool extensively utilized in cell biology and molecular genetics for the conditional destabilization of target proteins, facilitated by CRISPR/Cas9 gene knock-in technology [1, 2]. This mechanism capitalizes on the unique ability of the plant hormone auxin to rapidly degrade specific proteins bearing an AID sequence not only in non-plant cells like DLD1, HCT116, and HeLa but also in organisms like *Caenorhabditis elegans*, mouse, and yeast [3–6]. The AID assay furnishes a valuable means for probing protein functions and regulating protein expression in live cells. Its precise control over protein degradation addresses limitations of other methods such as RNAi gene silencing, which is both slow and less specific, lacking conditionality. The AID system, on the other hand, operates at the protein level, offering simplicity, rapidity, effectiveness, and reversibility. Central to this system is the interaction between the transport inhibitor response 1 receptor (TIR1), an F-box related protein, and the AID tag (available as 25 or 7 kDa), genetically fused to the protein of interest [7–9]. The half-life degradation of the tag, which is approximately 30 min, can be observed in a mammalian cell line expressing TIR1. Upon auxin binding to the TIR1 receptor, a conformational change occurs, allowing TIR1 to bind to the AID tag on the target protein. This interaction triggers the activation of the SCF (Skp1-Cullin-F-box) E3 ubiquitin ligase complex, comprising Skp1, Cul1, and RBX1. RBX1 associates with the E2 ubiquitin ligase, facilitating the ubiquitination of the protein of interest and its subsequent proteasomal degradation (26S proteasome). This system is advantageous as it leads to rapid degradation of the protein of interest in the presence of auxin, and its removal restores the phenotype [10, 11].

In our study, we employed the AID system as an innovative approach to investigate the mitotic functions of the replication licensing protein, CDT1. Through the AID system, we achieved precise and controlled inhibition of CDT1 during mitosis by inducing rapid degradation through auxin addition [12]. This strategy unveiled significant defects in mitotic spindle structure and chromosome alignment in treated cells. The system’s simplicity, control, and reversibility render it highly valuable for studying essential proteins and unraveling their roles in various cellular processes.

## Materials and reagents

1. Cas9-gRNA expression vector (e.g., pX330, PX458, PX459)
2. Parent repair template (a gift from Dan Foltz Lab)
3. Sense oligo for cloning of sg at BbsI site (5'-CACCG [sgRNA Target Sequence]-3')
4. Antisense oligo for cloning at BbsI site (5'-AAAC [reverse complement sgRNA Target Sequence] C-3')

**Biological materials**

1. DLD1-TIR1 cells, colorectal adenocarcinoma cell line (a kind gift from Andrew Holland, Johns Hopkins University, Baltimore, MD, USA)
2. One Shot^TM^ Stbl3^TM^ chemically competent *E. coli* (Thermo Fisher Scientific, catalog number: C737303)

**Reagents**

1. Dulbecco's modified Eagle's medium (DMEM) (Corning, catalog number: 10-013-CV)
2. Penicillin-Streptomycin 10,000 U/mL (Thermo Fisher Scientific, catalog number: 15140122)
3. Trypsin-EDTA, 1× (CORNING, catalog number: 25-052-CI)
4. Phosphate-buffered saline (PBS 1×) (CORNING, catalog number: 21-040-CV)
5. Effectene transfection agent (Qiagen, catalog number: 301425)
6. Dimethyl sulfoxide (DMSO) (Fisher Bio Reagents^TM^, catalog number: BP231-100)
7. Auxin (Indole-3-acetic acid, IAA) (Sigma-Aldrich, catalog number: 15148-2G)
8. Bovine serum albumin (BSA) (Sigma-Aldrich, catalog number: A7906)
9. Anti-GFP mouse monoclonal (Thermo Fisher Scientific, catalog number: A-11120)
10. Anti-Cdt1 rabbit polyclonal H-300 (Santa Cruz Biotech, catalog number: sc-28262)
11. Anti-Tubulin anti-mouse monoclonal DM1A (Santa Cruz Biotech, catalog number: sc-32293)
12. HRP-conjugated rabbit/mouse antibodies (Azure Biosystems, catalog numbers: AC2114 and AC2115)
13. SuperSignal^TM^ West Pico PLUS Chemiluminescent Substrate (Thermo Scientific, catalog number: 34580)
14. Plasmid Isolation kit (Qiagen, catalog number: 27104)
15. Gel Extraction kit (Plasmid and PCR clean-up kit) (Qiagen, catalog number: 28704)
16. Quick-DNA Microprep kit (Zymo Research, catalog number: D3020)
17. PCR Master Mix (2×) reagents (Thermo Scientific, catalog number: K0171)
18. GeneRuler 1 kb DNA ladder (Thermo Scientific, catalog number: SM0311)
19. TriTrack DNA loading dye (6×) (Thermo Scientific, catalog number: R1161)
20. T4 DNA ligase enzyme (NEB, catalog number: M0202S)
21. 10× buffer for T4 DNA ligase (NEB, catalog number: B0202S)
22. Bbs1 enzyme (NEB, catalog number: R0539S)
23. Ethidium bromide (Bio-Rad, catalog number: 161-0433)
24. Agarose HS (Denville Scientific Inc., catalog number: CA3510-8)
25. Ethylenediaminetetraacetic acid (EDTA) (Sigma-Aldrich, catalog number: 03620)
26. Sodium dodecyl sulphate (SDS) (Fisher Scientific, catalog number: BP166-500)
27. Tris-base (ChemCruz, catalog number: 77-86-1)
28. Ammonium persulfate (APS) (Sigma-Aldrich, catalog number: A3678)
29. TEMED (GE Healthcare, catalog number: 17-1312-01)
30. Acrylamide/Bis-acrylamide, 30% solution (Sigma-Aldrich, catalog number: A3574)
31. 6× Laemmli SDS sample buffer (Bioland Scientific LLC, catalog number: SAB03-01)
32. Pierce IP lysis buffer (Thermo Scientific, catalog number: 87787)
33. Halt protease inhibitor (100×) (Thermo Scientific, catalog number: 87786)
34. Acetic acid, glacial (Fisher Chemical, catalog number: A38-212)
35. Glycine 99% (Thermo Scientific, catalog number: A13816.36)
36. Sodium chloride (NaCl) (Fisher Chemical, catalog number: S271-1)
37. Magnesium chloride (MgCl_2_) (Sigma-Aldrich, catalog number: 208337-1KG)
38. S.O.C. medium (Invitrogen, catalog number: 15544-034)
39. Ampicillin sodium salt (Fisher Scientific, catalog number: 69-52-3)
40. Luria broth base (Miller's LB broth base), powder (Invitrogen, catalog number: 12795027)
41. LB agar (Lennox L Agar), powder (Invitrogen, catalog number: 22700025)

**Solutions**

1. 1 M Auxin (IAA) solution in Milli-Q water
2. 50× TAE buffer (see Recipes)
3. 1% agarose gel (see Recipes)
4. SDS-PAGE 10% resolving protein gel (5 mL)
5. SDS-PAGE 5% stacking protein gel (see Recipes)
6. Buffers for SDS-PAGE (see Recipes)
7. 100 mg/mL Ampicillin stock solution

**Recipes**

1. **50× TAE buffer**
  **Table:** 

Reagent	Quantity or Volume
Tris-base	242 g
Acetic acid	57.1 mL
EDTA	100 mL (0.5 M, 8.0 pH)
Milli-Q water	Adjust volume to 1 L
Total	1,000 mL
*Note: Dilute 50× TAE to 1× TAE (1:50) with Milli-Q water when making agarose gel and running the gel.*
2. **1% agarose gel (100 mL)**
  **Table:** 

Reagent	Final concentration	Quantity or Volume
Agarose powder	1%	1 g
1× TAE	N/A	100 mL
Ethidium bromide	0.5 µg/mL	2–5 µL
Total		100 mL
  - In a microwave-safe flask, add 1 g of agarose powder to 100 mL of 1× TAE buffer.
  - Microwave the flask in short 20–30 s intervals, swirling between each interval. Bring the mixture to a boil until the agarose has completely dissolved and the solution is transparent. Be careful as to not let the solution overboil and evaporate.
  - Allow mixture to cool before adding 5 µL of ethidium bromide for a 0.5 µg/mL concentration from 10 mg/mL stock. Swirl flask to mix.
  - Pour solution into casting gel tray and insert well comb. Allow gel to solidify at room temperature (RT).
3. **SDS-PAGE 10% resolving protein gel (5 mL)**
  **Table:** 

Reagent	Stock concentration	Volume
Milli-Q water	N/A	1.9 mL
Acrylamide mix	30%	1.7 mL
Tris-base	1.5 M, 8.8 pH	1.3 mL
SDS	10%	50 µL
APS	10%	50 µL
TEMED	N/A	2 µL
Total		5 mL
4. **SDS-PAGE 5% stacking protein gel (2.0 mL)**
  **Table:** 

Reagent	Stock concentration	Volume
Milli-Q water	N/A	1.4 mL
Acrylamide mix	30%	0.33 mL
Tris-base	1.0 M, 6.8 pH	0.22 mL
SDS	10%	20 µL
APS	10%	20 µL
TEMED	N/A	2 µL
Total		2 mL
5. **Buffers for SDS-PAGE**
  **Table:** 

Reagent	Running buffer (1×)	Transfer buffer (1×)
Glycine	14.4 g	2.9 g
Tris-base	3.03 g	5.8 g
SDS	1.0 g	0.33 g
Milli-Q water	1,000 mL	800 mL
Methanol	N/A	200 mL
Total	1,000 mL	1,000 mL

**Laboratory supplies**

1. 96-, 24-, 12-, and 6-microwell plates (Thermo Fisher Scientific, catalog number: 130188)
2. 15 mL conical Falcon tube (Fisher Brand, catalog number: 07-200-886)
3. 1.5 mL Eppendorf tubes (Fisher Scientific, catalog number: 02-682-002)
4. BioLite^TM^ cell culture treated dishes, 35, 60, and 100 mm Petri dish (Thermo Scientific, catalog numbers: 130180, 130181, and 130182)
5. Whatman^TM^ Uniflo^TM^ sterile PVDF syringe filters 0.22 µm (Cytiva, catalog number: 9913-2502)
6. Sterile polystyrene disposable serological pipettes (Fisher Brand, catalog number: 13-678-11E)
7. Flow cytometry tubes, Mini 35 µm (Olympus Plastics, catalog number: 28-154)
8. Sterile glass spreader

## Equipment

1. Centrifuge (Eppendorf, model: 5417C and 5804R)
2. Thermocycler (Eppendorf, model: GX2)
3. NanoDrop One (Thermo Scientific, model: Nanodrop One Spectrophotoshot Promo)
4. Flow cytometer (BD-FACS, model: Melody Cell Sorter)
5. Western blot developer (Azure biosystems, model: 600)
6. Agarose gel electrophoresis equipment (Bio-Rad, model: Mini-Sub Cell GT)
7. Protein gel electrophoresis equipment (Bio-Rad, model: Mini PROTEAN Tetra cell)
8. Spectrophotometer (Vinmax, model: 721-VIS)
9. Brightfield cell counter (DeNovix, model: CellDrop BF PAYG)
10. Inverted microscope equipped with a Yokogawa CSU-X1 spinning disc, an Andor iXon Ultra888 EMCCD camera, and a 60× or 100× 1.4 NA Plan-Apochromatic DIC oil immersion objective (Nikon, model: Eclipse TiE)

## Software and datasets

1. Broad Institute sgRNA design tool ( https://portals.broadinstitute.org/gpp/public/analysis-tools/sgrna-design). Alternatively, you can use CHOPCHOP (http://chopchop.cbu.uib.no/) or Crispor ( http://crispor.tefor.net/crispor.py)

## Procedure

- **Designing the guide RNA (gRNA)**
  1. Download the genomic DNA sequence from NCBI for your gene of interest. In this case, this is Cdt1. Go to website https://www.ncbi.nlm.nih.gov/gene/81620 and http://useast.ensembl.org/Homo_sapiens/Gene/Summary?g=ENSG00000167513;r=16:88803789-8880
  2. Open the full-length gene, including both introns and exons, and locate the translation start (ATG) and stop site (TGA) based on the position in the RefSeq (which is NCBI database).
  3. For N-terminal tagging, select and copy approximately 15 base pairs upstream of the ATG and approximately 150 base pairs downstream from the ATG. This sequence window will provide options to choose the best gRNA near to the point of modification.
  4. For C-terminal tagging, select and copy approximately 15 base pairs downstream of the TGA (stop codon) and approximately 150 base pairs upstream from the stop codon as stated earlier.
  5. Paste the selected sequence into the Crispor or/and CHOPCHOP sgRNA design tool, provided by Broad institute. Do not forget to select appropriate organism genome, which provides SG score, off-target, and valuable parameters.
  6. Download the results of the sgRNA design as a .txt file and import it into a spreadsheet.
  7. Review the sgRNA results obtained from the design tool and prioritize guides that cut within 50 base pairs after the ATG (for N-terminal modification) or close to Stop codon (for C-terminal modification), giving preference to those with none or fewer off-targets.
  8. Focus on specific columns, such as "Position of Base After Cut (1-based)," "sgRNA Target Sequence," "On-Target Rank," and "Off-Target Rank" to select guides with better scores.
  9. Pick at least three high-ranking guides for the cloning into SG backbone plasmid (Table 1).
    **Table 1.** Top rank sgRNA score table

sgRNA #	Orientation	sgRNA cut position	sgRNA sequence	sgRNA context sequence	* PAM* sequence	On-target rank	Off-target rank	Combined rank
1	Sense	168	GGCCCACCAGACACGTGCTG	GCCTGGCCCACCAGACACGTGCTGAGGAGG	AGG	3	5	1
2	Sense	173	ACCAGACACGTGCTGAGGAG	GCCCACCAGACACGTGCTGAGGAGGGGCTG	GGG	7	9	3
3	Sense	171	CCACCAGACACGTGCTGAGG	TGGCCCACCAGACACGTGCTGAGGAGGGGC	AGG	13	15	11
- **Cloning of SG sequence at Bbs1 site into pX330 vector to generate sgRNA constructs**
  1. Add the Bbs1 restriction enzyme overhang on 5′ and 3′ ends of the sgRNA sequence.
    Upstream cut: 5′ (CACCGG)GTCTTC, 3′ (CC)CAGAAG
    Downstream cut: 5′ GAAGAC(CT) 3′, 3′ CTTCTG(GACAAA) 5′
  2. Select sgRNA sequences based on the target region: order oligos for sense and antisense strands, including the sgRNA target sequence and its reverse complement (Table 2 and Table 3).
    Sense oligo: 5′-**CACCG** [sgRNA Target Sequence]-3′
    Antisense oligo: 5′-**AAAC** [reverse complement sgRNA Target Sequence] **C**-3′
    Order the oligos from your company of choice (e.g., IDT) and resuspend them in autoclaved Milli-Q water at a final concentration of 100 µM.
*Note: The sequences of SG for Cdt1 were reconfirmed using NCBI blast feature, and the first hit for all three sequences was the Cdt1 gene from Human.*
    **Table 2.** Primer list of top three sgRNAs with Bbs1-compatible cohesive ends

sgRNA#	sgRNA sequence	sgRNA with overhangs
1	GGCCCACCAGACACGTGCTG	Sense 5′ CACCGGGCCCACCAGACACGTGCTG 3′ antisense 3′ CCCGGGTGGTCTGTGCACGACCAAA 5’
2	ACCAGACACGTGCTGAGGAG	Sense 5′ CACCGACCAGACACGTGCTGAGGAG 3′ antisense 3′ CTGGTCTGTGCACGACTCCTCCAAA 5′
3	CCACCAGACACGTGCTGAGG	Sense 5′ CACCGCCACCAGACACGTGCTGAGG 3′ antisense 3′ CGGTGGTCTGTGCACGACTCCAAA 5′
    **Table 3.** Primer list of top three sgRNAs containing Bbs1-compatible cohesive end ready for ordering

sgRNA #	sgRNA sequence	Forward and reverse primer sequences
1	GGCCCACCAGACACGTGCTG	FP- 5′ ***CACCG*** GGCCCACCAGACACGTGCTG 3′ RP- 5′ ***AAAC*** CAGCACGTGTCTGGTGGGCC**C** 3′
2	ACCAGACACGTGCTGAGGAG	FP- 5′ ***CACCG*** ACCAGACACGTGCTGAGGAG 3′ RP- 5′ ***AAAC*** CTCCTCAGCACGTGTCTGGT**C** 3′
3	CCACCAGACACGTGCTGAGG	FP- 5′ ***CACCG*** CCACCAGACACGTGCTGAGG 3′ RP-5′ ***AAAC*** CCTCAGCACGTGTCTGGTGG**C** 3′
  3. Order the CRISPR/Cas9-EGFP sgRNA vector (pX330) and prepare it for cloning.
    **Plasmid details:** pX330, plasmid size: 8,484 bp ( https://www.addgene.org/42230/). Dissolve 8 µg of pX330 in 16 µL of Milli-Q water to achieve a final concentration of 0.5 µg/µL.
  4. Linearize the pX330 plasmid by BbsI enzyme.
    Milli-Q water 27.00 µL
    10× NE buffer 5 µL
    BbsI enzyme 2 µL
    pX330 backbone 16.00 µL (8 µg)
    Total 50 µL
  5. Incubate at 37 °C for 2 h.
  6. Inactivate the enzyme at 65 °C for 20 min.
  7. Purify the digested plasmid by gel purification using the gel extraction kit.
    - Prepare 1% agarose gel (see Recipes).
    - Mix 1 µL of ladder with 1 μL of 6× loading dye and 4 µL of Milli-Q water. Load the entire volume (50 µL) on gel. Mix 2 µL of uncut pX330 plasmid sample with 0.4 µL of 6× loading dye. Load the entire volume on gel.
    - Mix 2.5 µL of BsbI cut pX330 plasmid sample with 0.5 µL of 6× loading dye. Load the entire volume on gel.
    - Run gel at 90 V for 45 min.
- **sgRNA annealing and ligation**
  1. Prepare stock solutions of the gRNA primers.
    - The gRNA primer contains 120 µg of DNA and was diluted in 200 µL of Milli-Q water to achieve a concentration of 0.6 µg/µL. Dilute gRNA primer 1:100 to achieve a concentration of 6 ng/µL.
    - The reverse gRNA primer contains 170 µg of DNA and was similarly diluted in 200 µL of Milli-Q water to achieve a concentration of 0.85 µg/µL. Dilute reverse gRNA primer 1:100 to achieve a concentration of 8.5 ng/µL.
  2. Prepare the annealing reaction of each pair of oligos in annealing buffer as indicated in Table 4 and Table 5.
    **Table 4.** Annealing buffer

Reagent	Volume
Milli-Q water	45 μL
1 M HEPES	2.5 μL
1 M MgCl_2_	0.5 μL
2.5 M NaCl	2 μL
Total	50 μL
    **Table 5.** Annealing reaction

Reagent	Volume
Annealing buffer	48 μL
Sense oligo	1 μL (6 mg/mL)
Antisense oligo	1 μL (6 mg/mL)
Total	50 μL
  3. Run the reaction with the following conditions in a PCR machine:
    - 90 °C for 4 min
    - 70 °C for 10 min
    - Cool from 70 °C to 37 °C at a rate of 2 °C/min
    - 10 °C for 1 min
    - Hold at 4 °C
  4. Prepare ligation reaction with pX330 and sgRNA as indicated in Table 6. Prepare a negative control without sgRNA as indicated in Table 7.
    **Table 6.** 1:2 pX330:oligos ligation reaction

Reagent	Volume
Milli-Q water	4.68 μL
10× buffer for T4 DNA ligase	1 μL
Linearized pX330	2.82 μL
T4 ligase	0.5 μL
Annealed sgRNA oligos	1 μL (0.5893 ng) 1:200 dilution
Total	10 μL
    **Table 7.** Negative control

Reagent	Volume
Milli-Q water	5.68 μL
10× buffer for T4 DNA ligase	1 μL
Linearized pX330	2.82 μL
T4 ligase	0.5 μL
Total	10 μL
  5. Incubate ligation reactions at 16 °C overnight or RT for 2 h.
  6. Heat-inactivate the ligation reactions at 65 °C for 10 min (optional).
- **Transformation of ligated plasmid pX330 + gRNA and vector control**
  1. Set water bath to 42 °C.
  2. Thaw Stbl3 cells on ice for 10 min.
  3. Add 3 μL of the ligated plasmid or the vector control to 50 μL of Stbl3 cells.
  4. Incubate bacterial and DNA mixture on ice for 20 min.
  5. Heat shock in a 42 °C water bath for 45 s.
  6. Cool mixture on ice for 2 min.
  7. Add 400 μL of S.O.C. medium to mixture.
  8. Place in a 37 °C shaking incubator at 200 rpm for 1 h.
  9. Spin tube at 13,000 rpm for 1 min at RT to pellet.
  10. Remove 300 μL of media from the pellet. Resuspend pellet in the remaining volume of media.
  11. Plate 100 μL of cells on LB + Amp plate (Ampicillin working concentration 100 μg/mL) using sterilized spreader.
  12. Label plate and incubate in a 37 °C incubator overnight.
- **Screening of the clones**
  1. Examine plate for colony formation.
  2. On a new LB + Amp plate, use a marker to draw a 4 × 5 grid on the backside of the plate. Streak single colonies into their own spot on the grid.
  3. Incubate grid plate in a 37 °C incubator overnight.
  4. Next day, inoculate 4–5 colonies that grew on the grid into 10 mL of LB + Amp broth in 50 mL Falcon tubes to isolate the plasmid for sequencing.
  5. Place Falcon tubes in a 37 °C shaking incubator at 200 rpm overnight.
  6. Next day, isolate the plasmids using Plasmid Isolation kit (Qiagen) following manufacturer’s protocol.
  7. Send plasmid samples (Table 8) for sequencing to confirm the clone.
    **Table 8.** Sample for sequencing

Reagent	Volume
Putative pX330 candidate plasmid (20 ng/μL)	10 μL
U6 primer (10 ng/μL)	2 μL
Total	12 μL
- **Synthesizing Cdt1 gene and the AID+YFP tag into the repair template vector**
  We synthesized a DNA fragment containing 800 bp (between 500 and 1 kb) homology regions on each side of Cas9 cut site in the Cdt1 sequence; AID with YFP sequence was inserted in between these two homology arms (synthesized by Gene Universal). We then cloned this product into the vector HJURP using KpnI-HindIII restriction sites (a kind gift from Dr. Daniel Foltz, Northwestern University, Evanston, IL, USA). The PAM sequence within Cdt1 homology arms was mutated and replaced with synonymous codons without altering the protein sequence.
- **Transfect both sgRNA-cloned pX330 plasmid and the repair template (HJURP-AID-YFP) of Cdt1**
  1. Grow DLD1-TIR1 cells in complete DMEM media containing 1× penicillin-streptomycin and 10% FBS on a 60 mm plate. Incubate until they reach a confluency of 60%–70% on the day of transfection.
  2. In an Eppendorf tube, add the equimolar ratio needed (1 μg) of each plasmid containing Cdt1 sgRNA/Cas9 and Cdt1 repair template.
  3. Add 150 μL of EB buffer from the Transfection Reagent kit.
  4. Add 8 μL of Enhancer. Vortex for 1 s to mix.
  5. Incubate tube for 2–5 min at RT.
  6. Add 25 μL of Effectene transfection reagent. Vortex for 10 s.
  7. Incubate for 5–10 min at RT.
  8. Add 1 mL of incomplete DMEM media containing 10% FBS without penicillin-streptomycin to the Eppendorf tube. Gently mix by pipetting.
  9. Remove the complete media that is in the 60 mm plate. Wash the plate with 1 mL of incomplete media.
  10. Add the transfection tube contents to the cells drop by drop.
  11. Incubate at 37 °C with 5% CO_2_.
  12. After 6 h, add 2 mL of incomplete media to the plate.
  13. Incubate the plate for 24 h at 37 °C with 5% CO_2_.
  14. Remove the incomplete media from the plate.
  15. Add 4 mL of complete media to the plate.
  16. Incubate for 24–48 h at 37 °C with 5% CO_2_.
  17. When confluent, scale up cells into 100 mm plate.
  18. Five to seven days after transfection, harvest cells for sorting.
- **Harvesting cells for sorting**
  1. Collect the media from the cells growing in the 100 mm plate and filter with a 0.22 μm filter into a 15 mL Falcon tube. This will be used to make the conditioned media.
  2. In a new 15 mL Falcon tube, make conditioned media by combining 5 mL of the filtered media with 5 mL of complete media containing 20% FBS. Filter the conditioned media using a 0.22 μm filter.
  3. Wash the 100 mm cell plate with 1× PBS.
  4. Add 1 mL of 0.05% trypsin and incubate until cells detach.
  5. Add 9 mL of complete media to wash plate and detach cells.
  6. Collect cells into a 15 mL Falcon tube and centrifuge at 200× *g* for 5 min to pellet.
  7. Remove supernatant and resuspend cells with 5 mL of 1% FBS-1×PBS.
  8. Centrifuge cells at 200× *g* for 5 min.
  9. Remove supernatant and resuspend cells starting with 1 mL complete media.
  10. Perform cell count; then, add additional complete media such that there are 10 × 10^6^ cells per milliliter.
  11. In a 96-well plate, add 100 μL of conditioned media into each well for single-cell sorting.
  12. Sort single cells into 96-well plate using fluorescence-activated cell sorting with a flow rate of 1,000 events/second (evt/s).
- **Screening of monoclonal lines by confocal microscopy, PCR, and western blotting**
  1. Perform PCR.
    - Once cells are confluent in the 96-well plate, plate cells into a 24-well plate, followed by a 12-well plate, and then finally to a 6-well plate to ensure enough propagation of the single clones.
    - Once cells are confluent in the 6-well plate, trypsinize and collect cells of each clone.
    - Isolate genomic DNA of each clone using the Quick-DNA Microprep kit following manufacturer’s instructions.
    - Measure the DNA concentration and use as a template for PCR.
    - Design primers to check modification at targeted endogenous gene locus produced by CRISPR-Cas9 using PCR method (Figure 1 ).
      **Figure 1.**
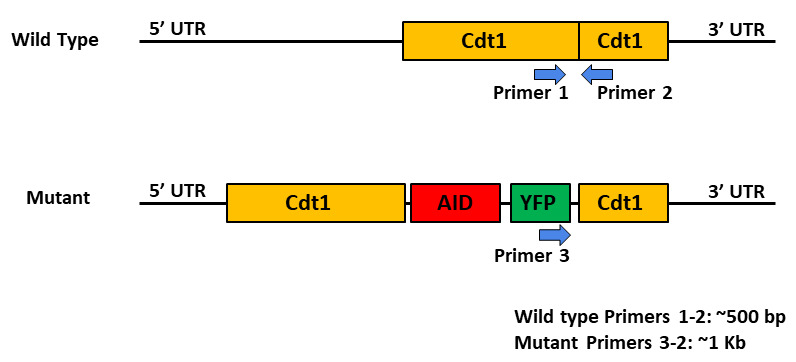
Strategy to confirm the homozygous and heterozygous knock-in events by PCR. Internal primers, as indicated, were designed for amplifying sequences with the Cdt1 gene (wildtype primers) and the AID-YFP insertion with Cdt1 gene (mutant primer 3).
    - Homozygosity and heterozygosity of the knock-in event in the clones will also be assessed by PCR. We ordered custom-made primers for the screening of clones from the company of choice (IDT).
    - Dissolve primers in Milli-Q water as indicated in Table 9 to achieve a concentration of 100 μM.
      **Table 9.** PCR reaction mixture for confirming homozygous and heterozygous clones

Reagent	Volume
PCR-Mix (2×)	10 μL (1×)
Primer 1	2.0 μL
Primer 2	2.0 μL
Primer 3	2.0 μL
Genomic DNA	X μL (25–50 ng)
Milli-Q water	Up to 20 μL
Total	20 μL
    - Run the reaction with the following conditions in a PCR machine for 25 cycles:
      95 °C for 5 min (initial denaturation)
      95 °C for 30 s (denaturation)
      55 °C for 30 s (annealing)
      72 °C for 1 min (extension time)
      72 °C for 5 min (final extension)
      Hold at 4 °C
*Note: Genomic DNA volume will depend on concentration. Annealing temperature will depend on primer T_M_.*
    - Analyze amplified DNA on a gel and check amplification profile. Control DLD-1 genomic DNA will produce a single band at 0.5 kb. For homozygous knock-in, the single band should be at 1 kb, while two bands at 0.5 kb and 1 kb would be seen for heterozygous knock-in.
    - For further confirmation of the knock-in events, purify the PCR product and sequence the product.
    - Clones that are PCR positive can be confirmed by confocal microscopy for GFP-positive cells.
  2. Western blot analysis of PCR-positive candidate strains identifies the best candidate, which expresses the AID-YFP tagged protein.
    - Grow DLD-1 cells only (for control) and AID-positive homozygous clone in 60 mm plates and incubate until confluent. Trypsinize cells and collect in 15 mL tubes.
    - Centrifuge cells at 200× *g* for 3 min to pellet and discard the supernatant.
    - Lyse cell pellet with 100 μL of Pierce IP lysis buffer containing 1 μL of halt protease inhibitor 100×. Keep on ice.
    - Incubate cells on ice for 10 min.
    - Centrifuge cells at 18,000× *g* for 20 min at 4 °C.
    - Collect the supernatant into new Eppendorf tubes and determine concentration.
    - Add 6× Laemmli SDS sample buffer to each sample of equal concentration.
    - Heat samples at 95 °C for 5 min.
    - Load samples of equal volume along with pre-stained protein ladder on the SDS-PAGE gel (see Recipes).
    - Transfer into membrane and block with 2% BSA in 1× PBS. Probe with primary anti-GFP and anti-Cdt1 antibodies followed by the secondary HRP-conjugated anti-mouse or anti-rabbit antibodies. Develop the blot using the chemiluminescent substrate kit following manufacturer’s instructions to confirm integration of AID.
    - Validate the GFP-positive cell clones via confocal immunofluorescence microscopy and confirm that GFP-AID is degraded in these cells after the addition of IAA. Prepare the AID clones for carrying out functional phenotypic analysis as carried out in Rahi et al. (2023) [12].
- **Validation of AID system**
  1. Split the homozygous GFP-positive clones into in 60 mm plates. DLD-1 cells will be used in a similar way for negative control.
  2. After 24–48 h of growing cells to 60%–70% confluency, treat cells with 500 μM of auxin (IAA) for the desired duration (0, 30, 60, and 120 min) to induce protein degradation.
*Note: The used concentration of auxin ranges from 50 to 500 μM, as obtained from previous published protocols, but this should be optimized for particular AID knock-in cell line. The best way to use is to start with the lowest concentration of auxin to prevent potential side effects while still effective in depleting the protein of interest. Treatment duration may vary depending on the AID-tagged protein such that protein levels are depleted without causing loss of viability.*
  3. Harvest the cells at various time points (0, 30, 60, and 120 min) after auxin treatment.
  4. Perform western blot using anti-tubulin antibody as a loading control or other appropriate protein analysis techniques to assess the protein degradation.
    Frozen stock of clones can be prepared in freezing media (90% FBS and 10% DMSO) and stored in liquid nitrogen.

## Data analysis

Analyze the protein degradation kinetics and determine the efficiency of the AID system in controlling protein expression. The AID assay provides a powerful tool for studying protein degradation and its regulatory mechanisms in mammalian cells. With proper optimization and controls, this technique can yield valuable insights into protein turnover and function.

## Validation of protocol

This protocol or parts of it has been used and validated in the following research article: Rahi et al. (2023). The Ndc80-Cdt1-Ska1 complex is a central processive kinetochore–microtubule coupling unit. J Cell Biol. (Figure 1, panel B, Figure S1, panel B) [12].

## General notes and troubleshooting

**General notes**

1. Make sure the PAM sequence is mutated in the donor plasmid/repair template.
2. Make sure that the TIR1+ mammalian cells (DLD1/HCT-116/HeLa/293T) being used are appropriate for the phenotypic or functional characterization experiments to be adopted.

**Troubleshooting**

Problem 1: Selection between heterozygous and homozygous clones.

Solution: For homozygous selection (biallelic), use two donor plasmids containing different selections (e.g., one plasmid with GFP and the other with m-Cherry). Cells can be sorted after insertion to confirm they contain two colors.

Problem 2: Difficulty inducing protein degradation by auxins.

Solution: Optimize concentration of auxins, starting with the lowest concentration and then increasing concentrations.

Problem 3: Auto/leaky degradation without the addition of auxins.

Solution: Use mini-AID (7 kD) or mutant form of AID (AID2) that is still able to properly bind to auxins (IAA/5-Ph-IAA).
